# DEVELOPMENT OF A SENSOR-ENHANCED CARE COORDINATION APPROACH TO FACILITATE AGING IN PLACE

**DOI:** 10.1093/geroni/igad104.0312

**Published:** 2023-12-21

**Authors:** Lori Popejoy, Blaine Reeder, Erin Robinson, Rachel Proffitt, Amy Vogelsmeier, Knoo Lee

**Affiliations:** University of Missouri, Columbia, Missouri, United States; Sinclair School of Nursing, University of Missouri, Columbia, Missouri, United States; University of Missouri School of Social Work, Columbia, Missouri, United States; University of Missouri, Columbia, Missouri, United States; University of Missouri, Columbia, Missouri, United States; University of Missouri, Columbia, Missouri, United States

## Abstract

Older adults living in rural areas of the country face unique challenges in accessing health services and community-based supports to facilitate aging in place (AIP). This is especially true for those with health concerns that have required care at a nursing facility. Re-establishing independence after a permanent discharge from a long stay nursing home can be difficult and puts strain on informal care networks. To address this, our team developed a novel approach to help older and disabled adults who were discharged from the Show-Me Home project, which supports clients permanently discharged from the nursing home. The Age-friendly Sustainable Smart and Equitable Technologies for Aging in Place (ASSETs for AIP) is a demonstration project funded by the Department of Health and Human Services. We are an interdisciplinary team of researchers and clinicians who use a strengths-based and client-driven approach to empower older and disabled adults for self-management of their disability, conditions, health, and well-being. Our care coordination team consists of a registered nurse, occupational therapist, and social worker, who assess health-related conditions, set goals, empower change, and monitor progress. For individuals enrolled in ASSETS for AIP, we rely on open-source smart home technologies and consumer-grade wearable devices to monitor activity patterns to identify changes in activity and health. Our care coordination team uses these data as well as client assessments to engage with clients to help them maintain independence. This model shows great promise in addressing the needs of older and disabled adults, thus enabling them to age in place.

